# Prediction of Responsiveness to Clomiphene Citrate in Infertile Women with PCOS

**Published:** 2019

**Authors:** Garima Sachdeva, Shalini Gainder, Vanita Suri, Naresh Sachdeva, Seema Chopra

**Affiliations:** 1- Department of Obstetrics and Gynaecology, Post Graduate Institute of Medical Education and Research, Chandigarh, India; 2- Department of Endocrinology, Post Graduate Institute of Medical Education and Research, Chandigarh, India

**Keywords:** Anti-mullerian hormone, Body mass index, Clomiphene, Hyperandrogenism, Polycystic ovarian syndrome

## Abstract

**Background:**

The purpose of the study was to evaluate the role of clinical, metabolic, hormonal and ultrasound features of women with PCOS in predicting the response to clomiphene citrate in treatment of infertility.

**Methods:**

A prospective observational study was done over a period of one year. A total of 164 women with PCOS related infertility were enrolled. They were treated with an incremental dose of clomiphene citrate starting with 50 *mg/day* to a maximum of 150 *mg* over 3 cycles. The response was recorded as either presence or absence of ovulation. Multiple logistic regression was used to analyze various clinical, metabolic, hormonal and ultrasound features in these women. Sensitivity and specificity of each of these parameters in predicting non-responsiveness (failure to ovulate with 150 *mg* clomiphene) were calculated.

**Results:**

Ferriman-Gallwey score, androstenedione levels, HDL, and cholesterol were found to be the independent predictors of non-responsiveness to clomiphene citrate. The overall best predictor of non-responsiveness to clomiphene citrate is Ferriman- Gallwey score (FG). FG score, with a cut off value of 15, had 73.9% sensitivity and 86.8% specificity in predicting non-responsiveness to clomiphene. BMI was the best anthropometric predictor of the non-responsiveness to clomiphene. Fasting insulin was the best metabolic predictor of the non-responsiveness to clomiphene. AFC was the best ovarian reserve marker as the predictor of the non-responsiveness to clomiphene (cut-off value of 11.75 with 73.9% sensitivity and 73.7% specificity).

**Conclusion:**

Ferriman-Gallwey score, androstenedione levels, and lipid profile are clinically useful parameters to predict which groups of PCOS women are unlikely to respond to clomiphene.

## Introduction

Polycystic ovary syndrome (PCOS) is a common problem in patients dealing with infertility (1). With increasing modernization, its prevalence is growing rapidly. PCOS falls in the category of WHO type II anovulation (norm-gonaddotrophic norm-estrogenic anovulation) and accounts for 85% of anovulatory females (2).

Clomiphene citrate is a triphenylethylene derivative which acts as an estrogen antagonist. It causes an increase in the release of gonadotropins from pituitary by blocking the negative feedback of estrogen and helps in the recruitment of the follicles (3). Clomiphene citrate (CC) is a drug that has been used for several years for combating PCOS related infertility (4). However, there is still an enigma as to why some PCOS women respond to the treatment while others do not. The prevalence of clomiphene resistance is as high as 15–40% (5).

Hyperandrogenism, obesity, insulin resistance, metabolic syndrome, and other endocrine abnormalities are proven associations or results of PCOS (6). But whether these factors also predict response to treatment in women with PCOS related infertility is still a dilemma. If the answer to this is yes, then the strength of these factors’ influence on the response is another question.

The study by Wenyan et al. (7) found AMH to be a significant predictor of non-responsiveness to clomiphene. Also, the study by Hamed et al. (8) demonstrated the role of patient’s BMI, testosterone, anti-mullerian hormone and ovarian volume in predicting response to clomiphene citrate treatment. A study by Johnson et al. (9) revealed BMI to be a predictor of response to ovulation in-duction agents. These studies give limited evidence about the factors influencing the response to clomiphene.

So, an attempt was made to analyze various clinical, metabolic, hormonal, and ultrasound parameters in predicting the response to clomiphene. Once predictors of ovarian responsiveness or non- responsiveness are found, a more specific treatment to our patients can be given. This will not only save the overall cost but will also save the precious time of our patients who come to us with a lot of hope.

## Methods

This prospective observational study enrolled 164 patients with PCOS related infertility from the infertility Out Patient Department (OPD) of a government hospital over the period of one year.

The study included infertile women with PCOS (based on Rotterdam’s criteria (10)) in age group of 18–40 years. Women on any insulin-sensitizing agent or lipid-lowering agent or even those having an endocrine disorder or anorexia nervosa/bulimia nervosa or with hypothalamic or pituitary dysfunction were excluded from this study.

All PCOS women desirous of pregnancy were evaluated only after taking a written informed consent. Relevant history was taken to rule out the exclusion criteria. The physical examination included blood pressure, weight in kilograms using a beam balance and height in upright posture without shoes using a stadiometer (to the nearest 0.5 *cm* was recorded). BMI was recorded based on the above measurements. Owing to the differences in body fat distribution between Asian and Western population, WHO expert committee in 2004 (11) has proposed BMI cut-offs for the Asian population which has been used in this study.

Waist circumference (WC) was measured midway between lower rib margin and the iliac crest in the mid-axillary line at the end of normal expiration (12). Hip circumference was measured with the measuring tape at the highest prominence of the buttocks and parallel to the floor (12). Waist and hip circumference were recorded after removing clothing from the area over waist and hip. The cut-off value of BMI was <23 *kg/m*^2^, and for waist circumference was 80 *cm*, and for the waist-hip ratio (WHR), the value turned out to be 0.81 based on the study conducted among the Asians (12).

Thyroid gland was examined for any enlargement, nodules or tenderness. The breast was examined for its characteristics and secretions. Signs of androgen excess like excessive hair growth, acne or alopecia were looked for. Excessive hair growth was evaluated by modified Ferriman and Gallwey (13) (FG) score.

The patients enrolled in the study were called on day 2 of their subsequent cycle for the investigations (FSH, LH, AMH, 17-hydroxyprogesterone levels (17 OHP), testosterone, androstenedione, 75 *gm* oral glucose tolerance test (OGTT), fasting insulin, fasting triglycerides, HDL (high density lipoprotein), LDL (low density lipoprotein), and cholesterol levels). Abdominal ultrasound was also done to rule out the fatty changes in the liver. The homeostasis model assessment of insulin resistance (HOMA-IR) (14), a surrogate marker of insulin resistance, was used in this study. Patients with HOMA-IR >2 were defined as having insulin resistance.

Baseline transvaginal scan (TVS) was done by the same observer using a Philips ultrasound machine, model IU22 (TVS probe frequency ranges 5–7 MHZ). Ovarian volume of each ovary was assessed by ellipsoid formula *i.e*. 0.52 X D1 (longitudinal) X D2 (oblique) X D3 (transverse) diameters. Mean ovarian volume was calculated by adding the volume of both ovaries and then dividing it by 2. Ovarian follicles in each ovary were examined and the total number of the follicles was counted by scanning each ovary from inner to the outer margin in longitudinal cross-section. Mean follicle number was calculated by adding the follicles of both ovaries and then dividing it by 2.

All these patients were treated with clomiphene citrate starting with 50 *mg/day* on day 2–5 of their cycle for 5 days. In case of failure of ovulation, the dose was increased by 50 *mg* in subsequent cycles to a maximum dose of 150 *mg* over 3 cycles.

Response to clomiphene citrate was assessed by ovulation. Transvaginal scan (TVS) was done by the same observer using a Philips ultrasound machine, model IU22. A scan was done starting from day 10 of the cycle and until reaching follicle size >18 *mm* or day 20 of the cycle. Patients were called after 2–3 days of development of dominant follicle to be checked for the rupture of the follicle.

Based on the ovulation pattern, these patients were divided into 2 groups, one who ovulated with maximum dose of 150 *mg* of clomiphene citrate and others who did not ovulate, as clomiphene citrate-resistant group.

The various parameters were compared between the clomiphene citrate-resistant and clomiphene citrate-sensitive groups.

The various parameters were recorded as Mean± SD. Normality of quantitative data was checked by measures of Kolmogorov Smirnov tests of normality and independent t-test or Mann Whitney U test was used based on whether data was normally distributed or not. Univariate and multiple logistic regression were used to study the various predictors of non-responsiveness to clomiphene citrate. Receiver operating characteristic (ROC) curve was used to assess the area under the curve (AUC), sensitivity, and specificity of each predictor. All statistical tests were two-sided and performed at a significance level of α=0.05. The analysis was conducted using IBM SPSS Statistics (version 24.0).

### Ethical consideration:

Informed consent was obtained from all individual participants included in the study. All procedures performed in studies involving human participants were in accordance with the ethical standards of the institutional research committee and with the 1964 Helsinki declaration and its later amendments or comparable ethical standards.

## Results

This study enrolled 164 women with PCOS related infertility. Of these, 88 (53.7%) were CC (clomiphene citrate) resistant and 76 (46.3%) were CC-sensitive. Baseline distribution and comparison between clomiphene citrate-resistant and sensitive group are given in table 1.

**Table 1.** Baseline mean distribution in the clomiphene sensitive and clomiphene resistant groups and respective p-values

**Parameters**	**Overall (MEAN±SD) (N=164)**	**Clomiphene resistant (MEAN±SD) (N=88)**	**Clomiphene sensitive (MEAN±SD) (N=76)**	**p-value**
**Age in years**	27.98±3.739	27.99±3.97	27.97±3.48	0.980
**BMI^1^ in *kg/m*^2^**	26.077±4.306	27.12±4.16	24.88±4.19	0.001
**Waist circumference in inches**	33.63±3.72	34.28±3.37	32.89±3.98	0.017
**Waist-hip ratio**	0.88±0.044	0.89±0.04	0.87±0.05	0.008
**Ferriman Gallwey score**	13.98±3.75	16.11±2.87	11.20±3.30	0.0001
**Testosterone (*nmol/l*)**	2.74±1.28	3.30±1.39	2.08±0.735	0.0001
**Androstenedione (*ng/ml*)**	2.97±1.36	3.54±1.53	2.31±0.723	0.0001
**OGTT^2^- FASTING (*mg/dl*)**	90.14±13.07	92.65±14.79	87.24±10.08	0.008
**OGTT^2^- 1 HOUR (*mg/dl*)**	148.02±35.22	158.33±36.05	136.08±30.325	0.0001
**OGTT^2^- 2 HOUR (*mg/dl*)**	130.73±29.37	137.57±29.94	122.82±26.67	0.001
**Fastingi (*mIU/L*)**	11.89±6.83	14.68±6.62	8.65±5.55	0.0001
**HOMA-IR^3^**	2.73±1.81	3.43±1.85	1.92±1.39	0.0001
**Serum triglycerides (*mg/dl*)**	133.11±50.91	146.35±60.93	117.19±29.71	0.0001
**Serum cholesterol (*mg/dl*)**	171.12±43.01	190.66±37.00	148.48±38.28	0.0001
**LDL^4^ (*mg/dl*)**	110.55±0.06	118.82±25.26	100.97±18.31	0.0001
**HDL^5^ (*mg/dl*)**	47.98±9.67	44.41±8.08	52.12±9.76	0.0001
**AMH^6^ (*ng/ml*)**	10.58±5.00	12.22±5.62	8.69±3.30	0.0001
**Mean ovarian volume in *cm*^3^**	12.52±3.07	13.65±3.26	11.21±2.23	0.001
**Mean AFC^7^**	11.81±3.17	13.19±3.07	10.21±2.47	0.000
**Baseline LH^8^ (*IU/l*)**	13.53±7.42	14.81±8.05	12.04±6.34	0.007
**Baseline FSH^9^ (*IU/l*)**	5.84±2.50	6.02±2.72	5.64±2.22	0.241
**LH^8^-FSH^9^ ratio**	2.48±1.18	2.66±1.22	2.27±1.11	0.035
**17 OHP^10^ (*ng/dl*)**	1.38±0.77	1.49±0.8	1.26±0.7	0.062

1-BMI- Body Mass Index, 2- OGTT- Oral Glucose Tolerance Test, 3- HOMAIR-Homeostatic Model Assessment Insulin Resistance, 4-LDL- Low Density Glycoprotein, 5-HDL- High Density Glycoprotein, 6- AMH- Anti Mullerian Hormone, 7-AFC- Antral Follicle Count, 8-LH- Luteinizing Hormone, 9-FSH- Follicle Stimulating Hormone, 10-17OHP- 17-Hydroxyprogesterone

To establish predictors of non-responsiveness to clomiphene citrate, logistic regression analysis was done.

In univariate regression analysis (Table 2) between CC-resistant and CC-sensitive PCOS women, BMI, waist circumference, Ferriman-Gallwey score, oral glucose tolerance test (OGTT)- fasting/1 *hr*/2 *hr* values, HOMA-IR, LDL, HDL, triglyceride, cholesterol, androstenedione, AMH, LH- FSH ratio, ovarian volume, and antral follicle count were found to be statistically significant. However, in multivariate logistic regression (Table 3), only Ferriman-Gallwey score, androstenedione levels, HDL and cholesterol were found to be the independent predictor of non-responsiveness to clomiphene citrate.

**Table 2.** Univariate regression analysis to predict the response to clomiphene using clinical, metabolic, hormonal and ultra-sonographic findings

**Variables in the equation**					

	**S.E. (Standard error)**	**p-value**	**Odd-ratio**	**95% C.I. for EXP(B)**	

				**Lower**	**Upper**
**BMI**^1^	0.041	0.001	1.140	1.053	1.235
**Waist circumference**	0.045	0.019	1.111	1.018	1.213
**Ferriman Gallwey score**	0.071	0.0001	1.628	1.417	1.870
**OGTT^2^- fasting**	0.013	0.009	1.034	1.008	1.061
**OGTT^2^- 1 *hr***	0.005	0.0001	1.020	1.010	1.030
**OGTT^2^- 2 *hr***	0.006	0.002	1.018	1.007	1.030
**HOMA-IR^3^**	0.121	0.0001	1.819	1.434	2.307
**LDL^4^**	0.008	0.0001	1.038	1.021	1.056
**HDL^5^**	0.022	0.0001	.901	.863	.940
**Serum triglyceride**	0.004	0.001	1.015	1.006	1.023
**Serum cholesterol**	0.005	0.0001	1.030	1.020	1.041
**Serum androstenedione**	0.227	0.0001	3.100	1.986	4.839
**AMH^6^**	0.042	0.0001	1.190	1.097	1.291
**Ovarian volume**	0.065	0.0001	1.352	1.190	1.536
**AFC^7^**	0.060	0.0001	1.244	1.106	1.399
**LH^8^-FSH^9^ ratio**	0.140	0.037	1.339	1.017	1.762

1-BMI- Body Mass Index, 2- OGTT- Oral Glucose Tolerance Test, 3- HOMAIR-Homeostatic Model Assessment Insulin Resistance, 4-LDL- Low Density Glycoprotein, 5-HDL- High Density Glycoprotein, 6- AMH- Anti Mullerian Hormone, 7-AFC- Antral Follicle Count, 8-LH- Luteinizing Hormone, 9-FSH- Follicle Stimulating Hormone

**Table 3.** Multivariate regression analysis to predict the response to clomiphene using clinical, metabolic, hormonal and ultra-sonographic findings

	**S.E.**	**p-value**	**Odd-ratio**	**95% C.I. for EXP(B)**	

				**Lower**	**Upper**
**BMI^1^ in *kg/m*^2^**	0.095	0.850	1.018	0.846	1.226
**Waist circumference**	0.115	0.492	0.924	0.737	1.158
**Ferriman Gallwey score**	0.113	0.0001	1.564	1.252	1.953
**Fasting blood sugar**	0.029	0.272	0.969	0.915	1.025
**HOMA IR^3^**	0.236	0.181	1.372	0.864	2.180
**LDL^4^**	0.023	0.932	1.002	0.957	1.049
**HDL^5^**	0.041	0.002	0.879	0.810	0.953
**Serum triglyceride**	0.007	0.837	0.999	0.986	1.012
**Cholesterol**	0.016	0.021	1.036	1.005	1.068
**Fatty liver present/absent**	0.673	0.682	0.759	0.203	2.839
**Serum androstenedione**	0.417	0.047	2.295	1.013	5.200
**AMH^6^**	0.081	0.322	1.083	0.925	1.269
**Ovarian volume**	0.116	0.237	1.147	0.914	1.440
**AFC^7^**	0.115	0.056	1.246	0.994	1.562
**LH^8^: FSH^9^ ratio**	0.288	0.060	1.720	0.978	3.024

Statistically significant AUC of each ROC curve in predicting non-response to clomiphene-citrate (CC) is depicted in table 4. The overall best predictor of non-response to clomiphene citrate in this study was Ferriman-Gallwey score with the maximum AUC of ROC curve. If the cut-off value of FG score is taken to be 15, it had 73.9% sensitivity and 86.8% specificity in predicting non-responsiveness to clomiphene.

**Table 4.** Predictors of non-response to clomiphene citrate

	AUC^*^	**P-VALUE**	**Cut-Value**	**Sensitivity**	**Specificity**
**Weight in *kg***	0.613	0.013	61.5	0.568	0.526
**BMI^1^ in *kg/m*^2^**	0.65	0.001	25.950	0.580	0.579
**Waist circumference**	0.623	0.007	33.750	0.602	0.579
**Waist-hip ratio**	0.624	0.006	0.885	0.580	0.671
**Ferriman Gallway score**	0.871	0.000	15.000	0.739	0.868
**Serum androstenedione**	0.763	0.000	2.480	0.682	0.684
**Testosterone**	0.784	0.000	2.41	0.705	0.724
**Serum triglyceride**	0.658	0.000	126.295	0.614	0.645
**Cholesterol**	0.784	0.000	168.465	0.705	0.711
**LDL^4^**	0.722	0.000	100.430	0.739	0.737
**HDL^5^**	0.732	0.000	48.320	0.671	0.670
**OGTT^2^- fasting**	0.605	0.020	88.500	0.580	0.605
**OGTT^2^- 1 *hr***	0.679	0.000	145.500	0.625	0.632
**OGTT^2^- 2 *hr***	0.646	0.001	129.500	0.602	0.605
**Fasting insulin**	0.759	0.000	10.77	0.67	0.67
**HOMAIR^3^**	0.757	0.000	2.215	0.659	0.658
**Mean ovarian volume**	0.755	0.000	11.785	0.739	0.724
**Mean AFC^7^**	0.779	0.000	11.75	0.739	0.737
**AMH^6^**	0.707	0.000	9.430	0.682	0.671
**Baseline LH^8^**	0.622	0.007	12.22	0.602	0.592
**LH^8^: FSH^9^ ratio**	0.605	0.02	2.240	0.602	0.605

AUC*- Area Under the Curve for Roc

**Figure 1.**
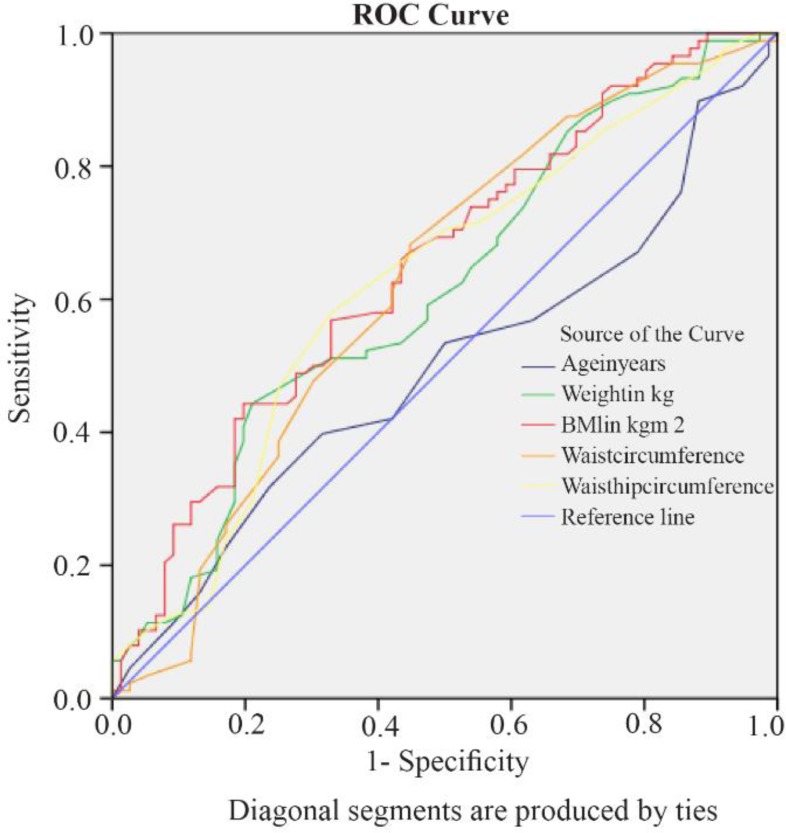
Receiver Operating Curve (ROC) of the Various Anthropometric Parameters

When considering the anthropometric parameters (age, weight, height, BMI, waist circumference, and waist-hip ratio), BMI was the best predictor of non-responsiveness to clomiphene with maximum AUC (Figure 1). If the cut-off value of BMI was taken as 25.950, it had 58% sensitivity and 57.9% specificity in predicting non-responsiveness to clomiphene.

**Figure 2.**
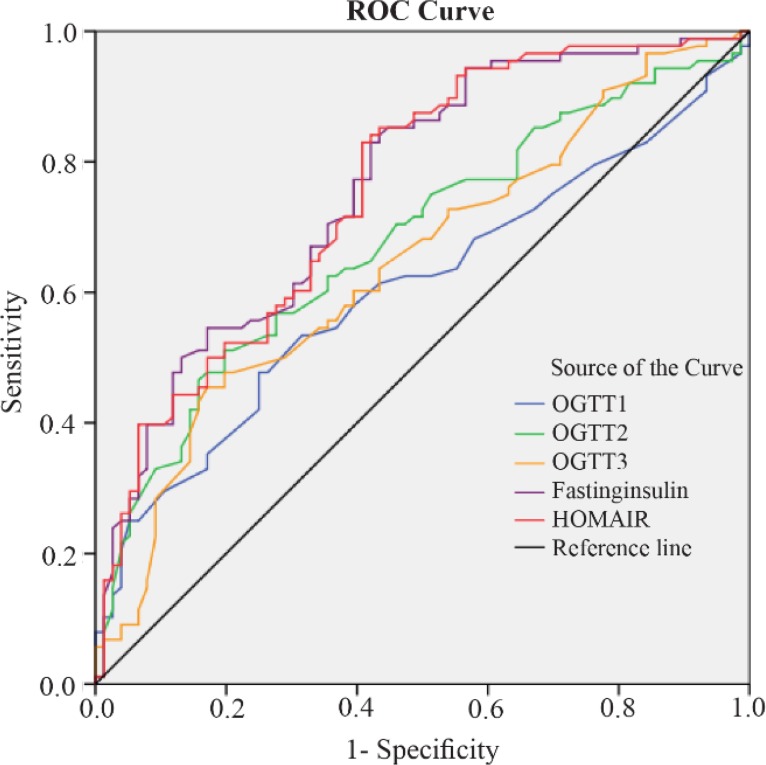
Receiver Operating Curve of the Various Parameters Used to Determine Insulin Resistance

Taking into account the parameters of insulin resistance (OGTT values, HOMA-IR, and fasting insulin), fasting insulin was the best predictor of the non-responsiveness to clomiphene. If the cut-off value of fasting insulin was taken as 10.77, it had 67% sensitivity and 67% specificity in predicting non-responsiveness to clomiphene (Figure 2).

**Figure 3.**
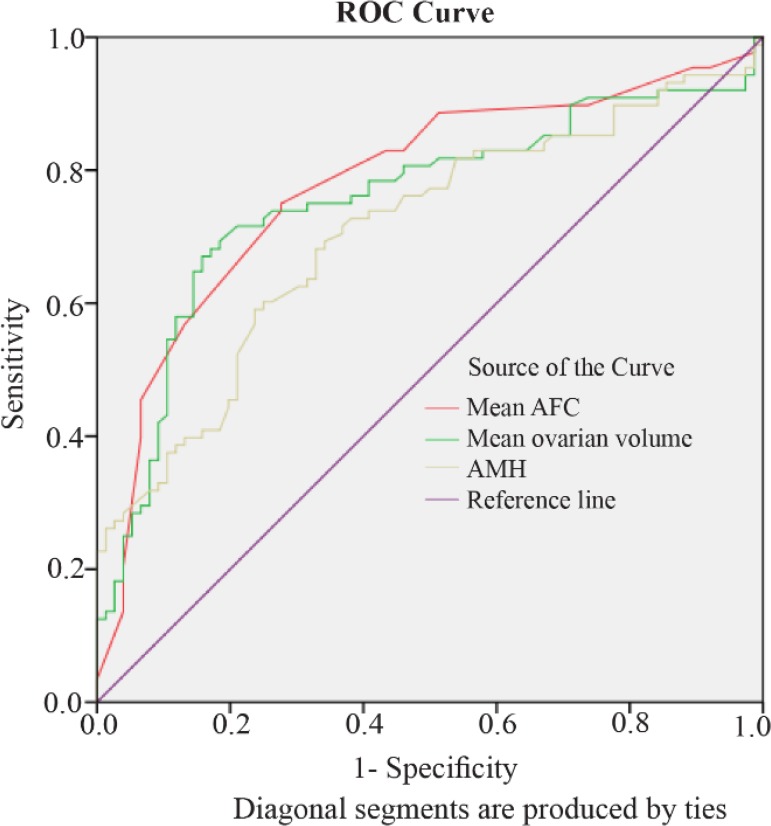
Receiver Operating Curve of the Various Parameters Used to determine ovarian reserve

Taking into account the parameters used for determining ovarian reserve (mean ovarian volume, mean AFC, and AMH), mean AFC is the best predictor of the non-responsiveness to clomiphene with maximum AUC of the ROC curve (Figure 3) (cut-off value of 11.75 with73.9% sensitivity and 73.7% specificity).

## Discussion

In this study, significant differences were seen in BMI, waist circumference, waist-hip ratio, parameters indicating hyperandrogenism (Ferriman-Gallwey score, testosterone, and androstenedione levels), parameters indicating insulin resistance (deranged oral glucose tolerance test, fasting insulin, and HOMA-IR), derangements in lipid profile (LDL, HDL, cholesterol, and triglyceride levels), ovarian reserve parameters (AMH, AFC, and ovarian volume), LH levels, and LH-FSH ratio amongst the clomiphene resistant and sensitive groups.

The results of the present study were based on the ovulation trend and not the conception. This is because conception is influenced by various other factors like endocrine dysfunction, tubal or uterine factors or male factors resulting in infertility. Clomiphene and other ovulation inducing agents primarily affect ovulation.

Hyperandrogenism (clinical and biochemical) proved to be a good predictor of clomiphene citrate resistance with high AUC curve value. The parameters for hyperandrogenism used in the study were Ferriman-Gallwey score, serum androstenedione, and testosterone. The study by Hamed et al. (8) and Imani et al. (15) manifested similar results. Increased androgens convert to estrone which causes negative feedback on hypothalamus and pituitary resulting in decreased FSH and anovulation. This is a possible explanation for increased hyperandrogenism which results in clomiphene resistance (16).

AMH, AFC, and ovarian volume are commonly used markers of ovarian reserve in clinical practice. The present study demonstrated higher AUC for AFC followed by mean ovarian volume and then AMH. These results differed from the study conducted by Nardo et al. (17), in which AMH was superior to FSH and AFC in predicting ovarian response. The study by Jayaprakasan et al. (18) also demonstrated that AMH and AFC either alone or in combination are good predictors of ovarian reserve. Also, the study by Wenyan Xi et al. (7) and Mahran et al. (19) found AMH to be a good predictor of clomiphene response. AMH is the hormone produced by primordial and small antral follicles (up to 6–8 *mm*). High AMH values result in anovulation by inhibiting FSH action and FSH receptor. This is the possible explanation for high AMH and AFC values resulting in poor responsiveness to clomiphene (20). So, in practice, it is always good to know the ovarian reserve of the patients before starting ovulation induction. However, more studies are required to establish the best ovarian reserve marker in predicting response to ovulation induction.

It is a known fact that PCOS is associated with insulin resistance and metabolic disturbances (21). However, no studies have yet been done to associate the metabolic profile of the patients in predicting response to clomiphene. In this study, BMI was a good predictor of non-responsiveness to clomiphene. With a cut-off value of 25.950, it had 58% sensitivity and 57.9% specificity in predicting non-responsiveness to clomiphene. Also, insulin resistance markers (OGTT values, HOMA-IR, and fasting insulin) can also help in predicting non-responsiveness to clomiphene. The best marker amongst these was fasting insulin. Hyperinsulinism results in hyperandrogenism by increasing LH induced androgen synthesis and decreasing sex hormone binding globulin (22). This explains increased clomiphene resistance in patients with hyperinsulinism. Also, deranged lipid profile, LDL >100.430 and cholesterol >168.465, had >70% sensitivity and specificity in predicting non-responsiveness to clomiphene. Thus, lifestyle modification and weight loss should always be offered to patients with obesity and metabolic disturbances. Weight loss results in decrease in cholesterol, triglyceride and LDL levels, improvement in insulin resistance, increase in sex hormone binding globulin and decrease in testosterone levels.

## Conclusion

Thus, based on this study, it is good to know the baseline hyperandrogenism, ovarian reserve parameters and metabolic profile of the patients before initiating clomiphene treatment. Screening the patients for these factors before starting ovulation induction treatment will help in providing appropriate counseling on the chances of success of treatment. Also, since the patients will save a lot of time and money, the degree of disappointment and frustration will naturally be less. Based on the results of this study, patients with severe hyperandrogenism, high AMH and AFC values and deranged lipid profile and insulin resistance should be given an option of alternative treatment after a short trial of clomiphene.
